# Infantile spasm, an unreported epilepsy form of CHD3 gene: A case report

**DOI:** 10.1097/MD.0000000000042801

**Published:** 2025-06-13

**Authors:** Tong Zhang, Wandong Hu, Huan Zhang, Ying Ren, Pingping Tian, Hongwei Zhang, Meng Wang, Song Su

**Affiliations:** a Epilepsy Center, Children’s Hospital Affiliated to Shandong University, Jinan, China; b Epilepsy Center, Jinan Children’s Hospital, Jinan, China; c Department of Neurology and Endocrinology, Children’s Hospital Affiliated to Shandong University, Jinan, China; d Department of Neurology and Endocrinology, Jinan Children’s Hospital, Jinan, China.

**Keywords:** CHD3 gene, heterozygous missense mutation, infantile spasms

## Abstract

**Rationale:**

CHD3 protein encoded by the CHD3 gene belongs to the second subfamily of the CHD family. The protein highly demonstrates in brain and is mostly in the developmental cortex. Its function is to promote the formation of late neural migration and cortex. Previously, CHD3 gene variants causing epilepsy are rare and only 1 case has been reported. The variation is a missense variant located at the C-terminal end of the deconvolution enzyme. However, there are no CHD3 gene mutations that have been reported to cause infantile spasms (IS) as yet. In the present study, we perform whole-exome sequencing (WES) in a patient of IS with neurodevelopmental disorders. This study suggests that CHD3 is potentially a candidate causative gene of IS.

**Patient concerns:**

Children was admitted to our hospital for a detailed evaluation of frequent seizures. Children have abnormal electroencephalogram and brain magnetic resonance imaging results, accompanied by developmental delay.

**Diagnoses:**

A genetic study using trio-WES confirmed the diagnosis of CHD3-related IS.

**Interventions:**

Patient accepted the treatment of antiepileptic drugs. One patient had seizure remission with a combination of adrenocorticotropin and topiramate.

**Outcomes:**

Whole-exome sequencing technology was used to determine the etiology of the children with IS. The patient had gained seizure-free, and the follow-up electroencephalography discharges were reduced.

**Lessons:**

This study confirmed that genetic testing provides a basis for the diagnosis of children with abnormal electroencephalogram and brain magnetic resonance imaging findings and developmental delay, and provides data supporting a future phenotype genotype correlation study. The CHD3 gene may be a new gene for IS, and the possibility of carrying a CHD3 gene variation should also be considered in children who present only with neurodevelopmental disorder without seizures.

## 1. Introduction

Infantile spasms (IS) is a refractory epileptic encephalopathy common in infancy, characterized by spastic seizures, highly disorganized electroencephalogram (EEG), and delayed or regressed psychomotor development. Approximately one-third of IS is hereditary, and dozens of genes have been found to be associated with IS, which can cause disease by affecting transcription factors, neuronal migration, signaling, neuronal metabolism and signaling pathways, as well as synaptic development and function.^[1,2]^ CHD3 gene (R1228W; 602120.0001) located on chromosome 17p13.^[3]^ Protein encoded by the CHD3 gene belongs to the second subfamily of the *CHD* family.^[4]^ The protein highly demonstrates in brain and is mostly in the developmental cortex. Its function is to promote the formation of late neural migration and cortex.^[5]^ When CHD3 gene expression is reduced, it leads to cortical layer abnormalities.^[6]^ CHD3 gene variants are now associated with both neurological and non-neurological abnormal phenotypes. Eising et al^[7]^ found CHD3 gene variants in a group of patients presenting with severe language and neurodevelopmental disorders. The group presents with language disorders, neurodevelopmental disorders, and appearance abnormalities, Mutations in the CHD3 gene is reported in the literature in association with clinically variable, neurodevelopmental disorders, which are characterized by language disorders, neurodevelopmental disorders, and appearance abnormalities, but epileptic phenotypes are rare, with only 1 case where the variation is a missense variant located at the C-terminal end of the deconvolution enzyme, but detailed clinical information, including seizure characteristics and treatment approaches, has not been specifically mentioned.^[7]^ Epilepsy has been described in only 1 patient with a CHD3 mutation, the only patient reported in the literature showed a missense variant located at the C-terminal end of the deconvolution enzyme. In this study, we report a patient with an epileptic phenotype carrying a novel heterozygous missense mutation c.3515G > A (p. Arg1172Gln) is found by using through whole-exome sequencing (WES). The child patient exhibits seizures, mental retardation, and peculiar facial features, the EEG shows high arrhythmias and clusters of seizures. Patient presents a marked widening of the cerebral fluid cavity on brain magnetic resonance imaging. Taking patient’s data together, it is suggested that CHD3 gene mutation may be a new pathogenic gene of IS.

## 2. Case presentation

This study was approved by the institutional review board of the authors. Informed consent was obtained from the patient for publication of this case report details.

The proband, male, at the age of 6 months due to “convulsion for 2 months” (March 2020). The clinical case involves a 6-month-old boy, the first child of nonconsanguineous parents. He was delivered by cesarean section at full term due to cephalopelvic disproportion. His birth weight was 3750 g and he did not suffer from hypoxia, asphyxia or infection during the perinatal period. There was no difficulty in feeding.

**Figure 1.**
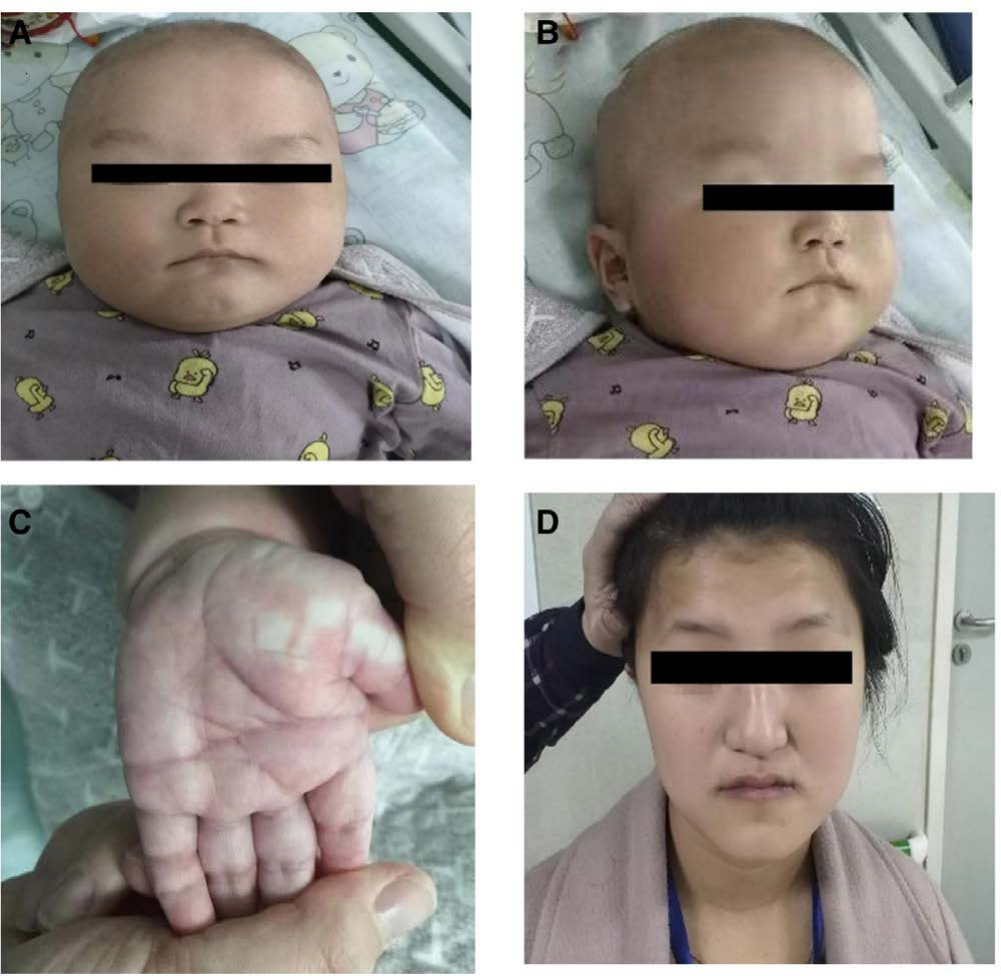
Appearance of the pediatric proband (6-mo-old) and the affected mother. (A and B) Wide distance between eyes (about 4 cm), low bridge of the nose, and low position of the ear; (C) palm with a straight line across in the left hand; (D) appearance of the patient’s mother: large head, frontal bossing, sparse eyebrows, anorthopia, long nose, and short philtrum.

Epilepsy onset was at the age of 4 months with the patient had a series of seizures that lasted a few seconds and took the form of more than 10 seizures per series, 3 to 5 seizures per day. At the age of 6 months, he cannot raise his head, turn over, sit or watch, but can listen to. The child is developmentally backward, with a body mass of 9 kg, large head, head circumference of 46 cm, bregma 4 cm × 4 cm, broad forehead, sparse eyebrows, wide eye spacing (about 4 cm), small cleft eyes, low nasal bridge, low ear position, left through palm (Fig. 1A–C), cardiopulmonary abdominal (−), low muscle tone in the limbs, and negative pathological signs. The child’s mother has a peculiar facial appearance (large head, protruding forehead, sparse eyebrows, squinting eyes, long nose, short human middle (Fig. 1D), delayed speech development (speech development started at 1 year and 7 months), and poor speech. She is optimistic and friendly. The child’s father and grandparents show no signs of developmental disorders, and there is no family history of epilepsy.

DNA was extracted from the peripheral blood of probands and their parents, and then a library was captured and constructed by IDT® xGen external probe hybridization for second-generation sequencing. The original data was > 10 G, capture efficiency was > 80%, coverage was 96%, and Q30 was ≥ 85%. Bioinformatics analysis and mutation screening were carried out according to the original data of the sequencer. The alignment rate of all the external data was > 98%, and the alignment efficiency of sequencing read number in the target capture area was > 75%. The average depth per person was > 100x, covering the exon regions of 20,000 human genes. The sequencing results were aligned with genome GRCh37 to detect sequence mutations. The statistical possibility of copy number of each exon was analyzed using DNA deletion and amplification (CNV) at the exon level by Weaver algorithm. The source of mutations (father, mother or new mutations) was determined by analysis of 3 family members, assisted with cosegregation and interpretation in the family, so as to eliminate mutation sites with unknown significance and remove false positive. The pathogenicity of the mutations was interpreted according to the ACMG guidelines. All clinically significant mutations were verified using first-generation Sanger sequencing (including probands and their parents). After the mother was detected to carry the mutation, first-generation Sanger sequencing was performed on the proband’s maternal grandparents.

**Figure 2.**
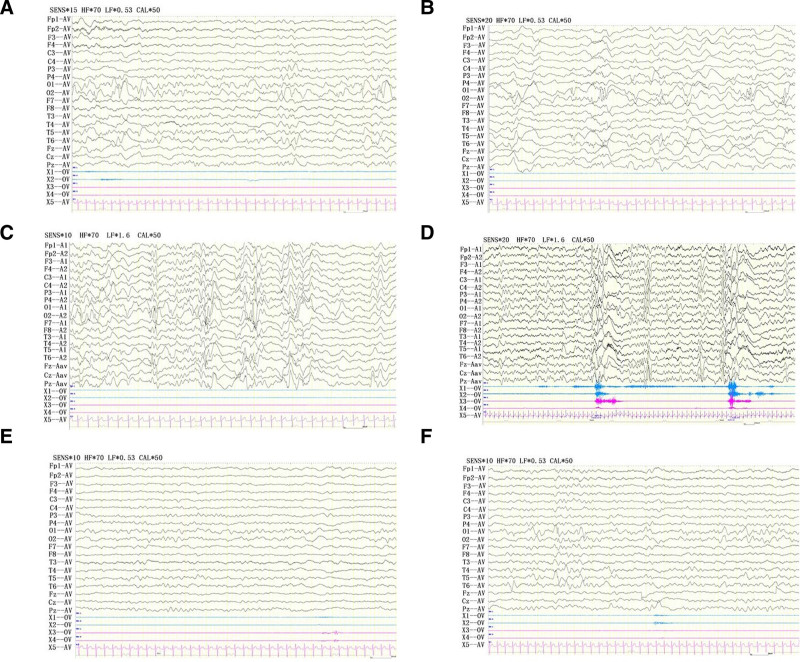
Electroencephalogram of the pediatric proband. Electroencephalogram at the first visit. (A) Mixed activity of low-medium amplitudes at a rate of 3 to 5 Hz primarily in bilateral occipital areas, with a fundamental symmetry between the left and right sides, and slowing of the basic rhythm; (B) a large number of multifocal spikes, sharp, spike-slow and sharp-slow waves intermittently of medium-high amplitudes primarily in bilateral occipital areas; (C) on the mixed slow wave background of diffuse mixed high-extremely high amplitudes, the presence of a large number of multifocal and extensive sharp, spike, polyspike, sharp-slow, spike-slow, and polyspike-slow complex waves of medium-extremely high amplitudes, showing a high degree of irregularity; (D) a series of spasms: extensive multi-phase slow waves of medium-high amplitude or the presence of slow waves with low amplitude and fast rhythm; (E) posttreatment electroencephalogram: at rest with closed eyes, θ activity of low-medium amplitudes at a rate of 5 to 6 Hz primarily in bilateral occipital areas, with a fundamental symmetry between the left and right sides, and no abnormality of the basic rhythm; (F) posttreatment electroencephalogram: a large number of sharp and spike waves of medium-high amplitudes, as well as sharp-slow and spike-slow waves at 2 to 3 Hz in bilateral occipital and posterior-central temporal areas, especially in the left side.

**Figure 3.**
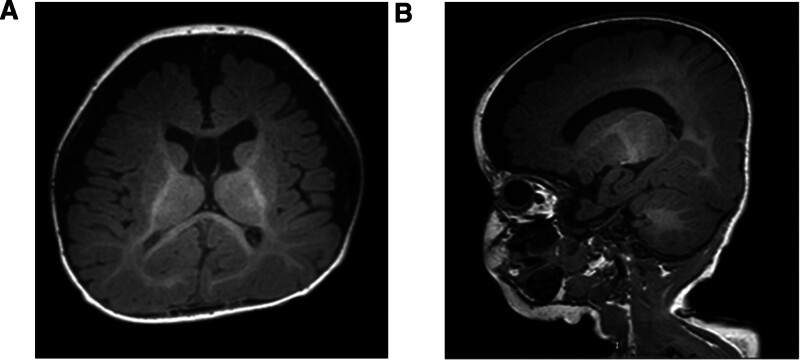
Magnetic resonance imaging of the pediatric proband. (A) Delayed myelination of white matter and significant widening of the cerebrospinal fluid space by axial T1-weighted brain MRI; (B) delayed myelination of white matter and apparent widening of the cerebrospinal fluid space by sagittal T1-weighted brain MRI. MRI = magnetic resonance imaging.

Video electroencephalogram (VEEG) (10–20 international system with 2000-Hz sampling rate; Nihon Kohden, Tokyo, Japan) of the patient at the age of 6 months is presented: slowed background activity, predominantly multifocal discharges in the back of the head, high arrhythmia during sleep, monitoring of several bouts of spasticity (Fig. 2A–D). Gesell developmental diagnostic scales: the proband has very severe developmental delays in adaptive, gross motor, fine motor, language, and personal-social development. The Peabody Motor Developmental Inventory indicates posture, movement, physical manipulation, grasping, and visual-motor integration are all < 1%. Cranial MR: the white matter of the brain is lagging behind in myelination and the fluid cavity is significantly widened (Fig. 3). Heart, genital, and joint color ultrasounds are normal. Visual evoked potentials and ophthalmologic fundus examinations are normal. Biochemistry, plasma lactate, plasma ammonia, screening for genetic metabolic disease, and chromosomal karyotype testing are negative.

**Figure 4.**
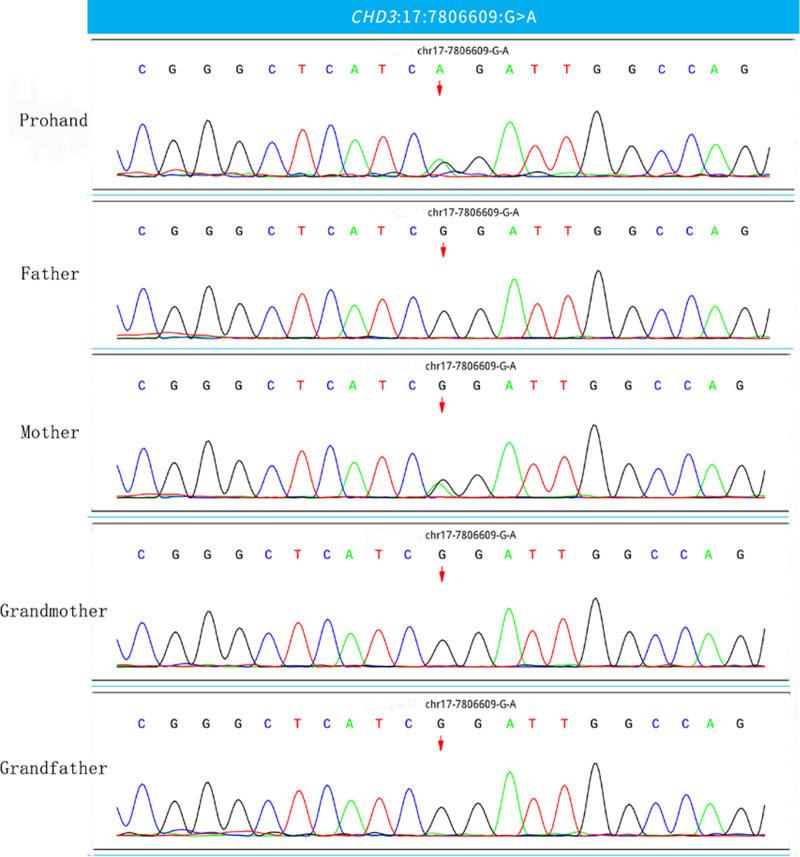
Family validation using Sanger sequencing in the infant with CHD3 gene mutation.

Family WES reveals a heterozygous missense variant c.3515G > A (p. Arg1172Gln) in the CHD3 gene (chr17:7806609), which results in the replacement of nucleotide G at position 3515 with A (c.3515G > A), with amino acid 1172 being changed from arginine to amino acid A (p. Arg1172Gln). This is from the mother with a milder phenotype. The child’s father and maternal grandparents do not carry the mutation (Fig. 4).

Treatment and follow-up: we give adrenocorticotropin (ACTH) treatment, 25 U/d × 2 weeks, topiramate oral 37.5 mg/d (4.2 mg/kg.d), 1 week seizure control. 2 weeks later, the child is discharged from the hospital when EEG shows bilateral posterior head discharge (Fig. 2E and F). At the last follow-up visit, the child is 10 months old, currently 4 months seizure-free. He can raise his head, roll over, but make no eye contact with people, cannot recognize people, cannot sit or crawl.

## 3. Discussion

Currently, many studies have shown that CHD3 gene variants can cause a variety of neurological and non-neurological abnormal phenotypes, but there are few reports related to epilepsy, and the case reported in this study is the first case in which CHD3 gene variants have been found in children with IS, and it is thought that the CHD3 gene may be a new gene for IS.

The CHD3 protein encoded by the CHD3 gene belongs to the second subfamily of the CHD family. Almost all CHD family proteins play a role in the development of the human nerve system. It has been found that CHD1, CHD2, CHD4, CHD7, and CHD8 genes are related to neurodevelopmental disorders.^[7–11]^ CHD3 protein and CHD4 protein belong to CHD subfamily II, and their sequence similarity High (about 70% amino acid identity), and the overall domain topology is similar. In brain, CHD4 is expressed throughout the cortex production process, and is present in neural stem/progenitor cells (NPCs) and neurons.^[6]^ In contrast, CHD3 is expressed later in late neuro migration and cortical formation, and decreased expression of CHD3 leads to cortical abnormalities. In NPCs, when CHD3 protein binds to nucleosome remodeling and deacetylase (NuRD) complex, CHD3-NuRD inhibits neurons activated by CHD4-NuRD.^[7,12,13]^

At present, CHD3 gene mutations are associated with abnormal phenotypes of the nerve system and nonneural system. Eising found CHD3 gene mutations in a group of patients with severe language and neurodevelopmental disorders. The group showed language impairment, neurodevelopmental disorders, and appearance abnormalities, but the epileptic phenotype was rare with only 1 case.^[7]^ Functional validation indicated that CHD3 gene missense variants may cause neurodevelopmental disorders by interfering with the chromatin remodeling activity of the encoded protein through effects on ATPase activity and chromatin remodeling capacity. The present child had very severe developmental delay, large head deformity, widened cerebrospinal fluid space, abnormal facial features (wide forehead, wide eye spacing, small cleft, low nasal bridge, and low ear position), and a left through palm, which is consistent with the CHD3 genetic phenotype reported by Eising. A previous study showed a widening of the cerebrospinal fluid gap in MR in 10 (33%) children with CHD3 gene mutation,^[7]^ and this is also the case in this case with cranial magnetic resonance imaging.

The genes of the reported cases are all new mutations. The CHD3 gene mutation in this case comes from the mother. The mother has a milder phenotype than the child, but she also has a special face, delayed speech development when she was young, and now she has a speech disorder, which is consistent with the variable phenotype and expression of the CHD3 gene. It has been verified that the proband’s maternal grandfather and grandmother do not carry the mutation, and the mother is considered to be a new mutation. In addition to the above neurodevelopmental disorders and facial deformities, the proband has a series of seizures at the age of 4 months. The VEEG shows a high degree of arrhythmia and a series of seizures, which is consistent with the performance of IS. We have improved the head MR and metabolism. Screening, etc, but have not found other causes of IS. The mutation site of the child is c.3515G > A (p.Arg1172Gln), located at the C-terminal end of the deconvolution enzyme, which is the same region as the mutation site of the phenotype of the reported epilepsy cases, so we think that the CHD3 gene may be the causative gene of infantile spasticity in the deceased. The possible pathogenic mechanism is that the mutation leads to a significant decrease in ATPase activity affecting chromatin remodeling activity leading to cortical abnormality, but the number of cases of CHD3 mutations is small, and more cases and functional validation are needed.

## 4. Conclusion

In this paper, by reporting a case of a child with CHD3 gene variation-associated neuro developmental disorder and IS, we found that the CHD3 gene may be a new gene for IS, and the possibility of carrying a CHD3 gene variation should also be considered in children who present only with neurodevelopmental disorder without seizures. To further investigate the relationship between CHD3 gene variants and IS and to better determine the prevalence of CHD3 gene variants, we should carry out further studies on the correlation between CHD3 variants and IS.

## Acknowledgments

The authors thank the parents of the children involved in this study and contributors to the study who are not included in the author list.

## Author contributions

**Data curation:** Wandong Hu.

**Investigation:** Huan Zhang.

**Methodology:** Pingping Tian, Meng Wang.

**Software:** Ying Ren.

**Writing – original draft:** Tong Zhang.

**Writing – review & editing:** Hongwei Zhang, Song Su.
